# SIRT6 polymorphism rs117385980 is associated with longevity and healthy aging in Finnish men

**DOI:** 10.1186/s12881-017-0401-z

**Published:** 2017-04-11

**Authors:** Katariina Hirvonen, Hannele Laivuori, Jari Lahti, Timo Strandberg, Johan G. Eriksson, Peter Hackman

**Affiliations:** 1 The Folkhälsan Institute of Genetics and the Department of Medical Genetics, University of Helsinki, Helsinki, Finland; 2 Medical and Clinical Genetics and Obstetrics and Gynecology, University of Helsinki, Helsinki, Finland; 3 Helsinki University Hospital, Helsinki, Finland; 4 Institute for Molecular Medicine Finland, University of Helsinki, Helsinki, Finland; 5 Institute of Behavioural Sciences, University of Helsinki, Helsinki, Finland; 6 Folkhälsan Research Centre, Helsinki, Finland; 7 University of Helsinki, Helsinki, Finland; 8 Helsinki University Central Hospital, Geriatrics, Helsinki, Finland; 9 Institute of Health Sciences/Geriatrics, University of Oulu, Oulu, Finland; 10 Department Chronic Disease Prevention, National Institute for Health and Welfare, Helsinki, Finland; 11 Department of General Practice and Primary Health Care, Helsinki University Hospital, Helsinki, Finland

**Keywords:** SIRT6, Longevity, Polymorphism, Aging

## Abstract

**Background:**

Sirtuin-6 (SIRT6) is involved in various crucial cellular pathways, being a key regulator of telomere structure, DNA repair, metabolism, transcriptional control and the NF-kappa B pathway. *Sirt6* knock-out mice have been reported to develop typical features of aging and senescence at the age of 2–3 weeks and die within 4 weeks. The aim of this study was to investigate whether sequence variations of SIRT6 are associated with aging and longevity in Finnish men.

**Methods:**

The sample of this study consisted of 43 longer-living and healthy males and 92 male control subjects who have died of natural causes at an average age of 66,6 (±4,1) years and who belonged to the Helsinki Birth Cohort Study (HBCS). Single nucleotide polymorphisms (SNPs) in the exons and their surroundings of the *SIRT6* were studied using direct PCR sequencing.

**Results:**

The SNP rs117385980 (C > T), situated 23 bases downstream of the exon 2 exon/intron border was found in heterozygous form in 1/43 longer-living healthy men (Minor allele frequency (MAF) 0,0116) and in 9/92 controls (MAF 0,0489). To replicate this finding, we studied a group of 63 healthy men at an average age of 83 years from the Helsinki Businessmen Study (HBS)–cohort. The heterozygosity of the same SNP was seen in 2/63 men from the HBS–cohort (MAF 0,0159). Fisher exact test was performed in our two combined study samples. The *P*-value for all samples combined was 0.07 and the odds ratio 3.53 (95% confidence interval 0.96–13.4).

**Conclusions:**

These results suggest an inverse association between the T allele of rs117385980 and longevity. The result needs to be confirmed in a larger study. It remains to be determined whether rs117385980 itself has an effect or if it is a mere genetic marker for some other yet undiscovered sequence variant causing a functional effect.

## Background

Despite extensive research, the molecular mechanisms of aging are incompletely understood. Aging is defined as an overall decline in the functional capacity of cells and organs caused by accumulated damage on the molecular level. It increases the risk for several unfavourable health outcomes including cardiovascular diseases, neurodegenerative disorders and cancer [1]. By studying and understanding determinants of longevity we will gain knowledge on the pathophysiology of several non-communicable age related diseases. This may potentially offer insights for new treatment strategies and therapies.

The role of NAD+–dependent proteins called sirtuins in longevity and lifespan was first detected in lower organisms. The first discovered sirtuin, Sir2 of *Saccharomyces cerevisiae* promotes longevity by preventing the formation of toxic extrachromosomal ribosomal DNA circles in yeast [2–4].

Sirtuin-6 (SIRT6) is one of the mammalian sirtuins (SIRT1-7). It is localized in the nucleus [5] and has mono-ADP-ribosylation–activity in addition to the NAD+–dependent deacetylation. SIRT6 is therefore involved in the regulation of multiple cellular pathways that affect cell senescence at the molecular level. The *sirt6* knockout mice develop features of aging at the age of 2–3 weeks and typically die within 4 weeks. Severe hypoglycemia, low levels of insulin-like growth factor 1 receptor (IGF-1), a curved spine, lymphopenia and loss of subcutaneous fat are observed and result in a progeroid-like syndrome [6]. *Sirt6* overexpression had opposite results; lifespan of male mice was extended by 15% due to a reduction in IGF-1 signalling in white adipose tissue [7].

SIRT6 maintains genomic stability by deacetylating telomeric amino acid residues. Deacetylation is also needed for chromatin compaction, transcriptional repression and DNA damage responses and preventing cellular senescence [8]. The lack of SIRT6 results in the formation of dysfunctional telomeres [9, 10]. Moreover, it is involved in multiple DNA repair systems where it assists the normal function of base excision repair [6] and double-strand break repair [11, 12]. It also regulates glucose metabolism by affecting both glycolysis [13] and gluconeogenesis [14]. Accumulation of triglycerides is observed in the absence of SIRT6 [15]. Recent studies have demonstrated SIRT6 having essential roles in heterochromatin silencing that contribute to cellular dysfunction associated with aging and disease that emerge especially in the absence of SIRT6 [16–18]. On top of everything else, it has been found that SIRT6 regulates also stem cell homeostasis and differentiation [19]. Taken together, SIRT6 is definitely needed for normal cell metabolism and preventing premature aging.

Despite different expression models and several studies of SIRT6 function the role of its genetic variations in humans is still poorly understood. The aim of this work was to investigate whether sequence variations of *SIRT6* are associated with aging and longevity in Finnish men. We report here the results of a study comparing a group consisting of longer-living and healthy men with male control subjects who died of natural causes at an average age of 66,6 (±4,1) years. To replicate the findings, we studied a group of healthy men at an average age of 83 years from the Helsinki Businessmen Study (HBS)–cohort [20].

## Methods

### Study population

The original study group consisted of men from the Helsinki Birth Cohort Study born in 1934–1944 in Helsinki. From the epidemiological cohort (*n* = 13 345), 2003 men participated in a clinical study in 2001–2004 (https://www.thl.fi/fi/web/thlfi-en/research-and-expertwork/projects-and-programmes/helsinki-birth-cohort-study-hbcs-idefix). On these subjects excellent phenotypic and genetic data is available. We identified a group consisting of 43 clinically healthy and still living men and 92 male controls who had died of natural causes at an average age of 66,6 (±4,1) years, at least 10 years earlier than the healthy males. The definition of ‘healthy aging’ includes both functional capacity and absence of major chronic diseases.

The study findings were replicated by using data from a group of 63 healthy men with an average age of 83 years from the HBS–cohort. The subjects themselves and their health status have been followed from the 1960s to present date for cardiovascular risks, mortality and health-related quality of life (HRQoL). Active and healthy aging was defined as followed; (1) participation in the postal questionnaire survey (to reflect social functioning); (2) no subjective memory problems (to reflect cognitive functioning); (3) feeling of happiness (to reflect psychological functioning and mental health); (4) no difficulties in overall activities of daily living (ADL, to reflect physical functioning); (5) no history of important chronic diseases (to reflect physical and mental health) [21].

### Sequencing

Genomic DNA was extracted from peripheral whole blood according to the manufacturer’s instruction, using a commercially available kit (QIAamp blood Maxi kit and DNeasy blood and tissue kit, Qiagen s.r.l. respectively). PCR amplification of the *SIRT6* exon 2 was performed using the forward primer 5′-CTCAGGCAAGACAGGGACAG-3′ and reverse primer 5′-TTAACGAACTTGGACCCTCC-3′. The total volume of the PCR reaction was 15 μL. The reaction mixture consisted of 50ng template DNA, 8μM of each primer, 3μl of 5xMyTaq Red buffer and 0,075μl MyTaq HS DNA Polymerase (Bioline, London, UK).

The PCR conditions for exon 2 were as follows: denaturation at 95 °C for 5 min followed by 30 cycles of denaturation at 95 °C for 30s; annealing at 65 °C for 30s; and extension at 72 °C for 30s. Final extension was at 72 °C for 7 min to ensure a complete extension of all the products. The PCR was performed on 2720 Thermal cycler (Applied Biosystems, Foster City, CA, USA). Amplified products were purified using ExoSAP-IT PCR Purification Kit (USB Corporation, Cleveland, OH, USA).

Primers and PCR conditions for other exons may be obtained upon inquiry.

The PCR products were sequenced using the Big-Dye Terminator v3.1 kit supplied by Applied Biosystems (ABI, Foster City, CA, USA). Reaction was performed on an ABI3730xl DNA Analyzer (Applied Biosystems at FIMM Genome and Technology centre Finland) according to the instructions of the manufacturer. The results were analyzed with Sequencher 5.0 software (Gene Codes Corporation, Ann Arbor, MI USA).

### Statistics

The Hardy-Weinberg equilibrium test was performed using the PLINK toolset for linkage analysis [22].

A Fisher exact test was first performed for HBCS samples to compare frenquencies of all discovered SNPs between healthy men and control men. Then the healthy and longer-living men from the two cohorts (https://www.thl.fi/fi/web/thlfi-en/research-and-expertwork/projects-and-programmes/helsinki-birth-cohort-study-hbcs-idefix, [21]) were combined for the comparisons of a specific SNP frequency between all longer-living and control men. A Fisher exact test was performed by PLINK for comparing frequencies. A *p*-value <0.05 was considered statistically significant.

## Results

Sequencing SIRT6 exons and their boundaries resulted in the discovery of one single nucleotide polymorphism, which was more abundant in the controls than in the longer-living. Rs117385980 (C > T), situated 23 bases downstream of the exon/intron border of exon 2 (19:4180588) was found in heterozygous form in 1/43 longer-living healthy men from HBCS (Minor allele frequency (MAF) 0.012) and 9/92 controls (MAF 0.049). Table 1 represents the results of the most significant associations between SIRT6 polymorphisms and longevity from a two-tailed Fisher exact test performed on HBCS samples (*n* = 135) and the type of polymorphisms. *P*-values of other SNPs remain relatively high and not significant.

**Table 1:** Results of association between SIRT6 polymorphisms and longevity from two-tailed Fisher exact test performed on HBCS samples (*n* = 135), prevalence odds ratios (ORs) and 95% confidence intervals (95% CIs) and the type of polymorphisms

OR	rs#	Position	95% CI	*p*-value	Type
2.37	rs192661013	4174661	0.27, 20.6	0.67	missense
1.54	rs350845	4174953	0.72, 3.31	0.36	splice region variant
3.36	rs352491	4177025	0.41, 27.8	0.44	intron variant
1.33	rs350844	4177054	0.63, 2.80	0.59	intron variant
0.70	rs11554579	4179253	0.11, 4.24	0.66	missense
1.33	rs7260071	4180693	0.63, 2.80	0.59	non coding transcript exon variant
0.34	rs352493	4180839	0.07, 1.55	0.21	missense
4.37	rs117385980	4180588	0.54, 35.1	0.18	intron variant

Rs117385980 was genotyped in the HBS-cohort and detected in 2/63 men (MAF 0.016) in heterozygous form. Genotypes of rs117385980 were in Hardy-Weinberg equilibrium in the study population. Healthy groups from two cohorts were combined and compared to controls. The *p*-value of two-tailed Fisher exact test performed for these was 0.07 whereas OR was 3.58 (95% CI: 0.96–13.4) (Table 2). Genotype frequencies were in agreement with Hardy-Weinberg equilibrium (*p* > 0.05).

**Table 2:** Results of association between SIRT6 and rs117385980 and longevity from two-tailed Fisher exact test performed on combined samples of HBCS and HB cohorts (*n* = 198)

rs#	Position	OR	95% CI	*p*-value	Type
rs117385980	4180588	3.58	0.96, 13.4	0.074	intron variant

Prevalence odds ratio (OR), 95% confidence interval (95% CI)

A power analysis conducted showed the power of 28.4% in our study.

## Discussion

These study findings suggest an inverse association between the T allele of rs117385980 and longevity in Finnish men. The T allele was present more often among subjects in the control group, i.e. those who died at an earlier age, than in the two groups of longer-living and healthy subjects combined. A trend of the CC genotype having a positive effect on the lifespan was thus observed.

*SIRT6* polymorphisms were studied in men from two carefully characterized Finnish cohorts. While the HBS cohort consisted of highly educated men, the participants in the Helsinki Birth Cohort Study represent a wide spectrum of socio-economic status (https://www.thl.fi/fi/web/thlfi-en/research-and-expertwork/projects-and-programmes/helsinki-birth-cohort-study-hbcs-idefix). Subjects’ age and health deviated remarkably from mean values in the general population; the longer-living groups are especially healthy for their age. We choose to study only men because of their life expectancy being 77,5 years, lower than that of women, being 83,4 years [23].

SIRT6 has turned out to be a notable gene associated with aging. It has previously been shown that SIRT6 SNP rs107251 genotypes of CC or CT had >5-year mean survival advantages compared to the TT genotype [24]. This study used a younger study population than ours; 65 years and older. It confirms our assumption of *SIRT6* having a specific influence on lifespan.

According to the 1000 Genomes project the frequency of the T allele of rs117385980 is 0,056 in Finland (http://www.ncbi.nlm.nih.gov/variation/tools/1000genomes/). This suggests that the CC genotype, which can be considered being the normal genotype in the population, could have a positive effect on the lifespan while the mutant CT genotype may have a zero or even negative effect. The possible positive effect of the CC genotype, due to a relatively large MAF is the norm of the population and becomes the normal and expected feature. Thus, the relatively small MAF of CT is generally interpreted so that CT has a negative effect and CC has no effect on the lifespan. Considering that rs117385980 is located reasonably close to an exon, it may affect splicing, which could in turn affect the function of the SIRT6-protein. This has not been shown here however. On the other hand, it is also possible that rs117385980 is a mere genetic marker for some currently unknown sequence variant, which affects the function of the protein.

The limitation of this study is the relatively limited number of participants.. To increase power of the analysis to 80%, as many as 427 cases and 371 controls would be required. Nevertheless, in a multifactorial trait such as longevity, a marked trend and obtaining a *p*-value of 0.07 is encouraging and commands further studies in larger cohorts. Thus possible coming steps would include expansion of the cohorts, verification of the findings in other cohorts and investigation of the possible functional role of rs117385980.

## Conclusions

Our results suggest an inverse association between the T allele of rs117385980 and longevity. The result needs to be confirmed in a larger study. It remains to be determined whether rs117385980 itself has an effect or if it is a mere genetic marker for some other yet undiscovered sequence variant causing a functional effect.

## Abbreviations

HBCS: Helsinki Birth Cohort Study
HBS: Helsinki Businessmen Study
MAF: Minor allele frequency
SIRT6: Sirtuin-6
